# Phenolic-Rich *Baccaurea angulata* Modulates Inflammatory Biomarkers of Atherosclerosis

**DOI:** 10.1155/2018/8406193

**Published:** 2018-11-05

**Authors:** Muhammad Ibrahim, Maryam Abimbola Mikail, Idris Adewale Ahmed, Radiah Abdul Ghani

**Affiliations:** ^1^Kulliyyah of Allied Health Sciences, International Islamic University Malaysia, Kuantan Campus, Kuantan, Malaysia; ^2^Department of Biomedical Science, Faculty of Science, Lincoln University College, Petaling Jaya, Malaysia; ^3^Department of Biotechnology, Faculty of Science, Lincoln University College, Petaling Jaya, Malaysia

## Abstract

**Purpose:**

Cardiovascular disease (CVD) is the leading and the most critical type of chronic disease. Atherosclerosis is the most common cause of CVD. Inflammation has been progressively acknowledged as a vital and central player in the pathophysiology of atherosclerosis. *Baccaurea angulata* is an underutilized fruit of the island of Borneo. It was obtained from Bau, Sarawak, Malaysia. In our previous studies, *B. angulata* did not only increase antioxidant enzyme activities, but also slowed the lipid peroxidation process in high-cholesterol-fed rabbits. It was hypothesized that *B. angulata* fruit would exert an anti-inflammatory effect. This study, therefore, aimed at evaluating and comparing the effects of three different *B. angulata* whole fruit (WF) juice doses on 11 serum inflammatory biomarkers of atherosclerosis.

**Methods:**

Thirty-five male New Zealand white rabbits were divided into seven groups (*n*=5). Group CH was fed 1% cholesterol diet only, group C1 was fed 1% cholesterol diet and 0.5 ml/kg/day *B. angulata* WF juice, group C2 was fed 1% cholesterol diet and 1.0 ml/kg/day *B. angulata* WF juice, group C3 was fed 1% cholesterol diet and 1.5 ml/kg/day *B. angulata* WF juice, group N was fed standard pellet only, group N1 was fed standard pellet and 0.5 ml/kg/day *B. angulata* WF juice, and group N2 was fed standard pellet and 1.0 ml/kg/day *B. angulata* WF juice for 12 weeks.

**Results:**

The administration of the various juices reduced the concentrations of induced serum inflammatory biomarkers.

**Conclusion:**

This protective effect of *B. angulata* fruit against cardiovascular risk might be due to its polyphenol content.

## 1. Introduction

Atherosclerosis, a systematic disease that manifests in the coronary circulation, has traditionally been highly correlated with dyslipidemia. When fats and lipids build up in and on the walls of large arteries, they restrict blood flow, resulting in thrombotic occlusion of the affected vessel [1]. Chronically expanding atherosclerotic lesions can lead to complete artery lumen occlusion and pose serious cardiovascular complications [2]. Fat accumulations in the arterial wall and hyperlipidemia have accordingly been the therapeutic targets in the prevention and treatment of atherosclerosis. Cholesterol-lowering drugs and a cholesterol-restricted diet are both common constituents of an antiatherosclerotic regimen [3].

Atherosclerosis begins when the inner cellular lining of the circulatory system, called the endothelium, becomes damaged in response to endothelial nitric oxide synthase (eNOS) dysfunction, monocyte attraction, activation of coagulation, and complement systems as well as increase in vascular permeability [4]. Low-density lipoprotein (LDL) crosses the damaged endothelium into the wall of the artery. Low-density lipoprotein builds up in enormous amounts and gets trapped within the arterial intima. This causes the white blood cells to leave the bloodstream and infiltrate the arterial walls to digest the LDL. Over many years, the toxic mess of cholesterol, white blood cells, and inflammatory cytokines combine to form plaque in the arterial wall [5]. Atherosclerosis is now widely accepted as an inflammatory disease involving chronic inflammation driven by immune responses to lipid deposition in the arterial wall. In addition to the accumulation of fat in the arterial walls, infiltrating macrophages, lymphocytes, and other inflammatory and immune cells that occur at such locations also significantly contribute to the pathology of this disease [6].

It is worthwhile mentioning that an inflammation is part of the complex biological process in which the vascular system helps fight harmful stimuli. At low levels (acute), inflammation is a lifesaver and acts as a protective attempt. Soon after any injurious stimuli, white blood cells move with urgent haste to the scene of infection to fight off the intruders and repair the damage [7]. However, there is a quiet, yet destructive, kind of inflammation that can seriously undermine the health: chronic inflammation—a silent trouble that does not only persist for a long time but also contributes to a host of other diseases such as atherosclerosis, if left unchecked [8]. Dietary factors as anti-inflammatory agents, however, are able to maintain health as well as reverse the progression of chronic diseases [9]. Therefore, current research efforts are focusing on inflammation as a new intervention strategy for the treatment of atherosclerosis. Several lines of evidence have supported the idea that circulating biomarkers of inflammation can be affected by diet composition [10].

Antioxidants play a favorable role on several mechanisms involved in combating atherosclerosis. Antioxidants are electron donors and thus prevent oxidation of LDL, one of the key events associated with atherosclerosis [10]. Antioxidants act as anti-inflammatory agents by interfering with inflammatory gene expression [11].

The results of earlier studies demonstrated the antioxidant properties of *Baccaurea angulata* fruit juice through the inhibition of LDL oxidation and enhancement of enzymatic antioxidants [12–17]. Furthermore, the beneficial effects of antioxidants on inflammation have been greatly reported. It was, therefore, hypothesized that *B. angulata* fruit would exert an anti-inflammatory effect. To explore this hypothesis, this present study aimed at evaluating and comparing the effects of three different doses of *B. angulata* whole fruit (WF) juice administered at 0.5, 1.0, and 1.5 ml/kg/day on 11 serum inflammatory biomarkers (cytokines) of atherosclerosis such as interleukin-1 (IL-1), interleukin-2 (IL-2), interleukin-6 (IL-6), interleukin-7 (IL-7), interleukin-8 (IL-8), interleukin-18 (IL-18), intercellular cell adhesion molecule-1 (ICAM-1), vascular cell adhesion molecule-1 (VCAM-1), C-reactive protein (CRP), tumor necrosis factor-*α* (TNF-*α*), and P-selectin in rabbits fed high-cholesterol diet. *Baccaurea* is a genus of flowering plant belonging to the family Phyllanthaceae. The genus comprises over 100 species, one of which is *Baccaurea angulata*. In English, the exotic fruit is usually called red angel starfruit, because of its resemblance with the starfruit (*Averrhoa carambola*). This fruit, however, is grown and very popular in the island of Borneo. It was obtained from Bau, Sarawak, Malaysia, and is locally called “Belimbing Dayak.”

## 2. Materials and Methods

### 2.1. Assay Kits

Rabbit interleukins (IL-1, IL-2, IL-6, IL-7, IL-8, and IL-18), ICAM-1, VCAM-1, CRP, TNF-*α*, and P-selectin kits were purchased from Cusabio (China).

### 2.2. Plant Materials

Fresh and fully matured *B. angulata* fruits were obtained from Bau, Sarawak, Malaysia. The fruits were morphologically identified and authenticated by an expert at the Forest Research Institute Malaysia (FRIM) and stored at −30°C.

### 2.3. Juice Preparation

The fruits without any physical damage were selected every day for juice preparation. The fruits were washed thoroughly with distilled water and then juiced with an electric fruit juicer exactly before consumption. The red skinned fruit has pinkish arils with a tangy taste and a sweet-tart flavour.

### 2.4. Cholesterol Diet Preparation

A 1% cholesterol diet was prepared by adding 40 g of analytical-grade pure cholesterol powder to each 3960 g of rabbit chow pellet (Bengy, Malaysia) (1% cholesterol, w/w, in food pellet). Cholesterol powder was first dissolved in 1600 ml of 99.9% chloroform and then mixed with rabbit chow pellets in a fume hood. After thorough mixing, the diet was transferred into a hot air oven, maintained at 37°C for chloroform evaporation. After about two days, cholesterol diet was stored in a chiller (2–8°C).

### 2.5. Animals and Study Design

Thirty-five male New Zealand white rabbits (Sapphire Enterprise, Seri Kembangan, Selangor, Malaysia) varying in weight from 2.45 ± 0.23 to 2.81 ± 0.27 kg on arrival were randomly housed in a controlled environment, maintained at 20 ± 1°C, 55 ± 5% humidity and 12-hour light/dark cycles. The rabbits were fed standard rabbit pellets and water ad libitum for 2 weeks to acclimatize. Following acclimatization, rabbits were then divided into seven groups of five rabbits each (*n*=5). Group CH was fed 1% cholesterol diet only, group C1 was fed 1% cholesterol diet and 0.5 ml/kg/day *B. angulata* WF juice, group C2 was fed 1% cholesterol diet and 1.0 ml/kg/day *B. angulata* WF juice, group C3 was fed 1% cholesterol diet and 1.5 ml/kg/day *B. angulata* WF juice, group N was fed standard pellet only, group N1 was fed standard pellet and 0.5 ml/kg/day *B. angulata* WF juice, and group N2 was fed standard pellet and 1.0 ml/kg/day *B. angulata* WF juice for 12 weeks. The foods (cholesterol diet and standard pellet) were restricted to 120 g. The dosage forms of *B. angulata* were administered to rabbits orally by sterile gavage. Fresh and clean water was provided either by open dish or nipple drinker. All experimental protocols were approved by the animal care and use committee of the Faculty of Medicine, International Islamic University Malaysia (IIUM) Kuantan Campus, Pahang (ID NO. IREC 05; Meeting No. 4/2012).

### 2.6. Blood Sampling and Analysis

Blood from rabbits that were deprived of food overnight [18] was obtained at week 0 and the end of the experiment (week 12). Blood samples were collected from the marginal ear vein. After centrifugation at 1800 g and 4°C for 15 minutes, serum was aliquoted and stored at −80°C until use. Each serum sample was assayed for inflammatory biomarkers of atherosclerosis.

### 2.7. Determination of Inflammatory Cytokines

Serum IL-1, IL-2, IL-6, IL-7, IL-8, and IL-18, ICAM-1, VCAM-1, CRP, TNF-*α*, and P-selectin were evaluated using the Cusabio (Hubei Province, China) ELISA kits. Analyses were done according to the manufacturer's instructions.

### 2.8. Statistical Analysis

Data were expressed as mean ± SD (*n*=5). The data were analyzed by ANOVA using Statistical Package for Social Sciences (SPSS) software (version 20.0, IBM), and the differences between the means were compared by multivariate analysis of variance (MANOVA) with Duncan's multiple range post hoc test. Statistical significance was considered at *p* < 0.05.

## 3. Results

The mean serum cytokine levels were obtained at weeks 0 and 12. According to the statistical evaluation, at the beginning of this study (week 0), there were no statistical differences in the concentrations of the serum cytokines between the groups of rabbits. Consequently, the mean values of the final results obtained (week 12) are presented in Table 1 for Interleukins-1, 2, 6, 7, 8, and 18 and Table 2 for ICAM-1, VCAM-1, CRP, TNF-*α*, and P-selectin.

### 3.1. Effects of *B. angulata* WF Juice Doses on Serum Interleukins-1, 2, 6, 7, 8, and 18

Serum IL-1 was significantly (*p* < 0.05) increased in the CH group. On the contrary, an elevated serum IL-1 level induced by feeding on high-cholesterol diet was reduced in the 0.5, 1.0, and 1.5 ml/kg/day *B. angulata* WF-treated groups (C1, C2, and C3) but with no dose-dependent effect. There was no statistically significant (*p* > 0.05) difference between groups N, N1, and N2 after 12 weeks of *B. angulata* WF administration.

High-cholesterol diet increased serum IL-2 concentrations in the groups CH, C1, C2, and C3. Statistically significant (*p* < 0.05) higher mean serum IL-2 level was seen in the CH group relative to the treatment groups (C1, C2, and C3). There was no statistically significant (*p* > 0.05) difference between groups N1 and N2. Group N, however, was significantly (*p* < 0.05) higher than groups N1 and N2.

No significant (*p* > 0.05) difference was found in the concentrations of serum IL-6 of groups CH, C1, C2, and C3. However, the IL-6 level of both C2 group and C3 group had no significant (*p* > 0.05) difference compared with that of group N. A dose-dependent pattern was found in the serum IL-6 of the C1, C2, and C3 groups. There appeared to be no significant (*p* > 0.05) difference between groups N, N1, and N2.

Serum IL-7 level in CH group was remarkably (*p* < 0.05) higher than those of the C1, C2, and C3 groups. Serum IL-7 showed no significant (*p* > 0.05) difference between groups N, N1, and N2. *Baccaurea angulata* WF dose-dependently reduced serum IL-7.

Serum IL-8 increased after feeding rabbits with high-cholesterol diet for 12 weeks. Administering *B. angulata* WF juice to high-cholesterol-fed rabbits lowered the serum IL-8 level in a dose-dependent manner. The concentrations of serum IL-8 of C1, C2, and C3 groups were significantly (*p* < 0.05) lower than that of the CH group. Likewise, that of the N group was significantly (*p* < 0.05) higher than those of the N1 and N2 groups.

High-cholesterol diet resulted in an increase in IL-18, which was ameliorated by treatment with *B. angulata* WF juices, but with no clear-cut dose-dependent pattern. Compared with the CH group given cholesterol diet only, all doses significantly (*p* < 0.05) decreased IL-18 of groups C1, C2, and C3. When comparing group N with the N1 and N2 groups, serum IL-18 levels were not significantly (*p* > 0.05) different between the groups.

### 3.2. Effects of *B. angulata* WF Juice Doses on Serum ICAM-1, VCAM-1, CRP, TNF-*α*, and P-Selectin

In contrast to the CH group, the concentrations of serum ICAM-1 significantly (*p* < 0.05) decreased in the *B. angulata* WF-treated groups (C1, C2, and C3). *Baccaurea angulata* WF dose-dependently decreased serum ICAM-1 in cholesterol-fed rabbits. No significant (*p* > 0.05) difference was found in the concentrations of serum ICAM-1 of groups N, N1, and N2.

Although no significant (*p* > 0.05) difference between CH, C1, C2, N, and N1 groups in serum VCAM-1 was observed, both groups C3 and N2, nevertheless, had a significant (*p* < 0.05) decrease compared with CH group.

There was no significant (*p* > 0.05) difference between the CH, C1, and C2 groups in their levels of serum CRP. Whereas, 1.5 ml/kg/day *B. angulata* WF significantly (*p* < 0.05) reduced serum CRP levels in the C3 group compared with CH, C1, and C2 groups. The magnitude of CRP increase was 9-fold greater in the CH group rabbits than in C3 group rabbits. A dose-dependent pattern was found in the serum CRP of the C1, C2, and C3 groups. No significant (*p* > 0.05) difference in CRP levels was observed between C3, N, N1, and N2 groups.

After 12 weeks of experimental study, high-cholesterol diet increased serum TNF-*α* concentrations in the groups CH, C1, C2, and C3. However, compared with groups C1, C2, and C3, the serum TNF-*α* level of rabbits in the CH group increased significantly (*p* < 0.05). The effect of *B. angulata* WF on serum CRP was found to be dose-dependent. There was no significant (*p* > 0.05) difference between groups N, N1, and N2.

The level of serum P-selectin in the CH group after 12-week feeding was raised significantly (*p* < 0.05) compared with C1, C2, and C3 groups. No significant (*p* > 0.05) difference was found in the concentrations of serum P-selectin of groups C1, C2, C3, and N after 12 weeks of *B. angulata* WF administration. Statistically significant (*p* < 0.05) lower mean serum P-selectin levels were seen in N1 and N2 groups relative to group N.

## 4. Discussion

The present study has demonstrated, in line with many recent studies, a link between high-cholesterol diet and inflammatory biomarkers of atherosclerosis. An etiologic role for cholesterol in atherosclerosis, though disputably, has been well established through laboratory and epidemiological studies. Cholesterol, a key causal factor in the genesis of atherosclerosis, has been viewed as one of the most important inducers of inflammatory biomarkers. Male rabbits fed high-cholesterol diet showed a high susceptibility to LDL oxidation and exhibited a significant elevation of high sensitive C-reactive protein (hsCRP), IL-6, and TNF-*α* in their blood [19]. In a study involving feeding rabbits a diet containing 1% cholesterol for 5-6 weeks, disturbances in the oxidant-antioxidant balance and an increase of inflammatory markers were reported [18]. The results of our study are consistent with previous observations. Our data showed that feeding rabbits a high-cholesterol diet for 12 weeks caused elevated inflammatory cytokines in serum.

An elevated level of IL-1, a prototypic proinflammatory cytokine, has long been thought to have a key role in the pathophysiology of atherosclerosis. Interleukin-1 causes the endothelial cells to produce more cytokines and adhesion molecules, thus leading to the recruitment of inflammatory cells [20]. We demonstrated that the administration of *B. angulata* WF juices (0.5, 1.0, and 1.5 ml/kg/day) exerted a reduction in the serum IL-1 level in high-cholesterol-fed rabbits (Table 1). Therefore, the use of *B. angulata* WF as a therapeutic plant-based food for the prevention of atherosclerosis progression could be interesting. Several possibilities may explain the wide health benefits of *B. angulata* WF, including the polyphenol contents which may contribute to its potentially protective role in atherosclerosis. Benn and colleagues [21] reported that diet-induced obese rats that were treated with polyphenol-rich blackcurrant extract showed a lower level of IL-1 than the controls. Likewise, pretreatment with water extract of polyphenolic *Ziziphus jujuba* fruit (Jujube) suppressed the expression of IL-1*β* in cultured murine macrophages [22].

Similarly, serum IL-2 levels were also reduced with all doses (0.5, 1.0, and 1.5 ml/kg/day) of *B. angulata* WF (Table 1). Interleukin-2, a cytokine produced by activated T-lymphocytes, has long been known as one of the major contributors to atheromatous plaque formation [23]. Given the fact that there is no safe level of drug use and individuals taking oral immunosuppressive medications put themselves at even higher risk for adverse health effects, *B. angulata* WF could be one of the viable options for the prevention of inflammatory process underlying atherosclerosis. Similar to *B. angulata* WF, a 46% reduction of stimulated IL-2 was reported in blood after oral intake of medicinal mushroom *Agaricus blazei* Murill in healthy volunteers [24].

Another interesting finding from this study shows that the elevated serum IL-6 level induced by feeding high-cholesterol diet was dose-dependently reduced in the *B. angulata* WF-treated groups of rabbits (Table 1). In a study by Jung and colleagues [25], the mRNA level of IL-6 was downregulated by pressure extract of ginseng. Thus, the present study is important for its contribution to data available on health benefits of underutilized fruits. Many researchers have studied the in-depth nutrition facts and possible use of many well-known fruits, including their effects on cardiovascular health. However, very few underutilized fruits have been scientifically studied. These versatile health benefits exerted by *B. angulata* WF may be partly due to the fact that phenolic compounds which act as excellent antioxidants indirectly contribute to anti-inflammatory action. *Baccaurea angulata* WF that possessed the potential to minimize the generation of free radicals and displayed high antioxidant properties and protective effect against lipid peroxidation, a promoter of the atherosclerotic event [16], also interferes with the inflammatory stage of atherosclerosis. Our results are in accordance with a previous experimental study which showed that polyphenol-rich *Euterpe oleracea* Martius (Arecaceae), commonly known as açaí, possessed both antioxidant and anti-inflammatory properties through the reduction of lipid peroxidation, boosting of antioxidant enzymes, and inhibition of proinflammatory cytokine production [26, 27].

Moreover, oral administration of *B. angulata* WF juices (0.5, 1.0, and 1.5 ml/kg/day) dose-dependently reduced experimentally induced master regulator of T-cell development, IL-7 (Table 1). It has been shown that IL-7 induces inflammation in the endothelium and recruits monocytes/macrophages to the endothelium, thus playing a critical role in atherogenesis [28]. In our present study, *B. angulata* WF appeared to be effective in reducing this protagonist of atherosclerosis.

In addition, in the scientific literature, there are enough facts to support beyond any doubt the involvement of IL-8 in the recruitment of inflammatory cells into atherosclerotic plaques [29]. An experimental study [30] has found that an aqueous extract of the fruit of *Crataegus pinnatifida* var. major decreased the concentration of IL-8 in atherosclerotic rats fed a high-fat diet. This present study with an underutilized fruit, to our knowledge, is the first to report a reduction in IL-8 (Table 1), which plays a crucial role in leukocyte trafficking and activation, in high-cholesterol-fed rabbits [29]. Administering *B. angulata* WF juices to high-cholesterol-fed rabbits lowered the serum IL-8 level in a dose-dependent manner. Interleukin-8 represents one of the key enhancers of the inflammatory microenvironment of the insulted vascular wall [29]. Thus, the present study justifies the use of *B. angulata* WF in the traditional system of medicine for the prevention and treatment of atherosclerotic inflammatory response.

Furthermore, IL-18 has also been linked to atherosclerotic events, owing to its expression by macrophages in human plaque and relation to plaque instability [31]. The importance of this cytokine as proatherogenic in mice models of inflammation has been demonstrated by Elhage and colleagues [32]. The authors reported reduced atherosclerosis, in spite of increased serum cholesterol, in IL-18 deficient apoE(−/−) mice. In this study, we observed that the elevated levels of serum IL-18 induced by high-cholesterol feeding were ameliorated (Table 1) after treatment with *B. angulata* WF juices (0.5, 1.0, and 1.5 ml/kg/day). The decrease in IL-18 expression which is in agreement with another study [30] suggests that *B. angulata* WF may be a promising option for the treatment and prevention of atherosclerotic vascular disease.

In addition, ICAM-1 is the major adhesion molecule in the development of atherosclerosis. Intercellular cell adhesion molecule-1 plays an important role in the recruitment of leukocytes to sites of atherosclerotic lesions [33]. Involvement of ICAM-1 in the progression of atherosclerosis in the ApoE-deficient mice model has been previously reported [34]. Our data also showed that the 12-week treatment with *B. angulata* WF exerted a distinct anti-inflammatory effect as demonstrated by reduced serum ICAM-1 levels in *B. angulata* WF-treated rabbits (groups C1, C2, and C3) compared with group CH (Table 2). *Baccaurea angulata* WF dose-dependently decreased serum ICAM-1 in cholesterol-fed rabbits. This finding is in accordance with a recent study which showed that *Piper sarmentosum* extract inhibited the expression of ICAM-1 in high-cholesterol-fed rabbits [35].

In addition to exerting an ICAM-1-reducing effect, *B. angulata* WF also effectively reduced another immunoglobulin-like adhesion molecule, the VCAM-1 (Table 2). The molecule that is encoded by the VCAM-1 gene has long been postulated to play a more critical role in the development of atherosclerosis. As previously reported, proinflammatory cytokine levels were increased concurrently with elevated circulating VCAM-1 in atherosclerosis [36]. The exact mechanism of action for *B. angulata* WF against this family of adhesion molecules (ICAM-1 and VCAM-1) is uncertain, but it is thought that *B. angulata* WF exerted its adhesion molecule-lowering effect in high-cholesterol-fed rabbits perhaps due to inhibition of c-Jun N-terminal kinase (JNK) pathway. The mechanism of this effect is similar to that of adhesion molecule inhibitors, which was originally designed to hinder adhesion molecules' biological activities by inhibiting JNK pathway [37].

Serum level of CRP is a determinant of atherosclerosis severity. C-reactive protein contributes significantly to atherosclerosis owing to its predictive influence for cardiovascular events [38]. In our present study, *B. angulata* WF caused a reduction of serum CRP levels in the *B. angulata* WF-treated rabbits (Table 2). Several animal and clinical studies have demonstrated the antiatherosclerotic effects of polyphenol-rich plant products, in part by lowering the CRP level [39]. Therefore, we suggest that *B. angulata* WF may be useful for the therapeutic treatment of CRP-associated atherosclerosis. Interestingly, it has been suggested that CRP modulates the process of atherosclerosis by inducing endothelial dysfunction and uncoupling endothelial nitric oxide synthase (eNOS) mRNA expression [40]. Thus, *B. angulata* WF was able to decrease serum levels of induced CRP, which may improve endothelial function.

Several lines of evidence support the crucial involvement of TNF-*α* in the pathogenesis of atherosclerosis. A study demonstrated that in apolipoprotein E-deficient (apoE^−/−^) mice, levels of TNF-*α* expression are closely associated with lesions in atherosclerosis-prone sites [41]. In this study, as compared with group CH, there was a dose-dependent decrease in serum TNF-*α* levels of C1, C2, and C3 groups (Table 2).

P-selectins are lectin-like adhesion glycoproteins that function as cell adhesion molecules which mediate leukocyte rolling on the surfaces of activated endothelial cells. The effect and role of P-selectin in the development of atherosclerosis are vital. A 50–75% reduction in atherosclerotic lesion size has been demonstrated in P-selectin-deficient apolipoprotein E-knockout mice [42]. According to a study, extracts from Ginkgo biloba leaves inhibit the inflammatory process in mice through interfering with the expression of P-selectin [43]. In the present study, *B. angulata* WF juices ameliorated serum P-selectin levels in groups C1, C2, and C3 as compared with the CH group (Table 2). Therefore, our study provides evidence that *B. angulata* WF may also reduce progression of atherosclerosis.

Similarly, *B. angulata* WF appeared to decrease serum cytokines in groups N1 and N2 compared with group N. The levels of IL-2, IL-8, and P-selectin in Group N was, however, significantly higher (*p* < 0.05) than those in groups N1 and N2. Our results confirmed that a properly balanced diet is essential to reduce risk factors for chronic diseases such as CVD. Maximum health benefits could, therefore, be achieved with normocholesterolemic diet and *B. angulata* WF, which is likely due to its phenolic constituents.

## 5. Conclusion

The present study demonstrated that *B. angulata* WF juice had very pronounced anti-inflammatory effects. It lowered the concentrations of serum inflammatory biomarkers of atherosclerosis in high-cholesterol-fed rabbits. Similar lowered serum inflammatory biomarkers were observed in rabbits fed standard pellet (normocholesterolemic groups) and were administered *B. angulata* WF juices. With hypercholesterolemic diet, 1.5 ml/kg/day *B. angulata* WF juice appeared to be more effective than both 0.5 and 1.0 ml/kg/day *B. angulata* WF juices in reducing the cytokines. These results suggest that the anti-inflammatory effects of *B. angulata* WF might be due to its phenolic constituents or might be partly due to its antioxidant properties.

## Figures and Tables

**Table 1:** Effects of *B. angulata* WF juice doses on serum Interleukins-1, 2, 6, 7, 8, and 18.

Groups	IL-1 (pg/ml)	IL-2 (pg/ml)	IL-6 (pg/ml)	IL-7 (pg/ml)	IL-8 (pg/ml)	IL-18 (pg/ml)
CH	72.33 ± 6.17^a^	228.92 ± 11.10^a^	127.27 ± 10.12^a^	10.43 ± 1.14^a^	191.62 ± 13.56^a^	79.36 ± 7.30^a^
C1	47.94 ± 2.82^b,c^	123.01 ± 10.02^b^	120.63 ± 7.64^a^	2.48 ± 0.11^b^	165.63 ± 10.04^b^	8.62 ± 1.17^c^
C2	57.75 ± 4.63^b^	137.19 ± 7.04^b^	98.65 ± 7.21^a,b^	1.98 ± 0.56^b^	145.83 ± 9.00^c^	15.18 ± 3.97^c^
C3	39.77 ± 2.53^c^	102.72 ± 9.39^b^	95.58 ± 6.56^a,b^	1.72 ± 0.32^b^	53.47 ± 9.73^e^	27.58 ± 4.09^b^
N	37.31 ± 5.87^c^	205.86 ± 11.37^a^	86.60 ± 8.15^b,c^	1.57 ± 0.27^b^	78.67 ± 7.12^d^	15.78 ± 2.02^c^
N1	43.74 ± 4.29^b,c^	119.78 ± 7.37^b^	77.68 ± 11.50^c^	2.44 ± 0.09^b^	55.17 ± 8.00^e^	14.25 ± 2.16^c^
N2	38.09 ± 5.03^c^	103.33 ± 9.12^b^	70.27 ± 6.54^c^	1.96 ± 0.56^b^	53.15 ± 8.95^e^	13.90 ± 2.29^c^

Values are given as mean ± SD (*n*=5). ^a,b,c,d,e^Values not sharing a common superscript letter within the same column differ significantly at *p* < 0.05. CH: 1% cholesterol diet; C1: 1% cholesterol diet and 0.5 ml/kg/day *B. angulata* WF juice; C2: 1% cholesterol diet and 1.0 ml/kg/day *B. angulata* WF juice; C3: 1% cholesterol diet and 1.5 ml/kg/day *B. angulata* WF juice; N: standard pellet; N1: standard pellet and 0.5 ml/kg/day *B. angulata* WF juice; N2: standard pellet and 1.0 ml/kg/day *B. angulata* WF juice; IL: interleukin.

**Table 2:** Effects of *B. angulata* WF juice doses on serum ICAM-1, VCAM-1, CRP, TNF-*α*, and P-selectin.

Groups	ICAM-1 (ng/ml)	VCAM-1 (pg/ml)	CRP (pg/ml)	TNF-*α* (pg/ml)	P-selectin (pg/ml)
CH	21.49 ± 4.07^a^	352.83 ± 16.98^a^	47.48 ± 5.16^a^	123.71 ± 13.06^a^	30.36 ± 2.40^a^
C1	7.88 ± 0.96^b^	308.49 ± 11.79^a,b^	44.42 ± 6.99^a^	37.16 ± 4.11^b^	15.05 ± 1.73^b^
C2	4.56 ± 0.68^c^	337.67 ± 11.95^a,b^	36.78 ± 4.78^a^	27.76 ± 3.89^b^	15.50 ± 3.21^b^
C3	2.78 ± 0.16^c,d^	173.82 ± 9.83^b^	5.75 ± 1.56^b^	13.64 ± 2.19^b^	12.43 ± 2.45^b^
N	1.08 ± 0.26^d^	278 ± 12.22^a,b^	14.45 ± 3.64^b^	6.45 ± 0.47^c^	14.30 ± 1.05^b^
N1	0.93 ± 0.20^d^	265.76 ± 10.54^a,b^	5.96 ± 1.38^b^	7.38 ± 1.46^c^	8.90 ± 0.73^c^
N2	0.95 ± 0.18^d^	198.71 ± 9.49^b^	6.38 ± 0.73^b^	6.68 ± 0.23^c^	7.57 ± 0.75^c^

Values are given as mean ± SD (*n*=5). ^a,b,c,d^Values not sharing a common superscript letter within the same column differ significantly at *p* < 0.05. CH: 1% cholesterol diet; C1: 1% cholesterol diet and 0.5 ml/kg/day *B. angulata* WF juice; C2: 1%. cholesterol diet and 1.0 ml/kg/day *B. angulata* WF juice; C3: 1% cholesterol diet and 1.5 ml/kg/day *B. angulata* WF juice; N: standard pellet; N1: standard pellet and 0.5 ml/kg/day *B. angulata* WF juice; N2: standard pellet and 1.0 ml/kg/day *B. angulata* WF juice; ICAM-1: intercellular cell adhesion molecule-1; VCAM-1: vascular cell adhesion molecule-1; CRP: C-reactive protein; TNF-*α*: tumor necrosis factor-*α*.
